# Acquired Cystic Kidney Disease: A Hidden Complication in Children on Chronic Hemodialysis

**DOI:** 10.7759/cureus.24365

**Published:** 2022-04-22

**Authors:** Sabeeta Khatri, Irshad Bajeer, Ali Asghar A Lanewala, Muhammad Farid, Seema Hashmi

**Affiliations:** 1 Pediatric Nephrology, Sindh Institute of Urology and Transplantation, Karachi, PAK; 2 Radiology, Sindh Institute of Urology and Transplantation, Karachi, PAK

**Keywords:** ultrasonography, end stage kidney disease, renal replacement therapy, solitary cysts, acquired cystic kidney disease

## Abstract

Objective

To determine the frequency of acquired cystic kidney disease (ACKD) in children on chronic hemodialysis.

Material and methods

In this single-center cross-sectional study, 150 children were included who were on chronic hemodialysis for six months. Ultrasound was done to see the renal cysts. Cystic changes that could not fulfill the criteria for ACKD were also noted and analyzed.

Results

The mean age was 14.5 ± 3.5 years, of these 63 (42%) were males. Acquired cysts were detected in 53 (35%) of the patient and 18 patients (12%) had solitary cysts. The distribution of these entities was similar across all age groups. The underlying etiologies in the descending order were unknown 64 (43%), stone disease 31 (21%), each of the congenital anomalies of the kidney and urinary tract, and glomerulonephritis 23 (15%), and others nine (6%). A higher frequency of ACKD was detected in the children on renal replacement therapy for more than two years (33 out of 53 children, 63% with a p-value of 0.004).

Conclusion

The ACKD was found in one-third of our hemodialysis children and its frequency increases with the duration of hemodialysis. This percentage may not reflect the true prevalence as there is a lack of consensus on the definition of ACKD. Periodic assessment of chronic kidney disease patients for the development of ACKD especially on chronic hemodialysis is required to reduce the morbidity.

## Introduction

Acquired cystic kidney disease (ACKD) was first reported in 1977 by Dunnill et al. in patients on long-term hemodialysis [1]. It is characterized by the development of bilateral renal cystic changes in individuals without a history of inherited or congenital cystic kidney disease and is observed as a complication in chronic kidney disease (CKD) patients, particularly those receiving long-term hemodialysis or peritoneal dialysis [2]. Irrespective of the etiology of renal disease, any insult causing the decreased number of nephrons initiates the cascade of events due to the release of certain growth factors and activation of proto-oncogenes. These agents lead to compensatory hypertrophy of the remaining nephrons which over the years culminates in the cystic transformation of kidneys [3].

The reported incidence of ACKD shows wide variation due to a lack of consensus about the definition and different imaging modalities used to visualize the cysts. In adult literature, the incidence of ACKD varies from 10% to 95% [4]. So far there are only four reports that have described ACKD in children with kidney disease. Based on the available data incidence varies from 21% to 46% in children on dialysis and frequency increases with a longer duration of dialysis [5]. As in adults, ACKD has been found to occur in pre-dialysis children and in patients receiving any form of renal replacement therapy (RRT) including hemodialysis, peritoneal dialysis, and kidney transplant [6]. Spontaneous regression of cystic changes in native kidneys after renal transplant has been described. However metastatic tumors are not uncommon due to lowered immunosuppression [7].

Majority of the patients with ACKD are usually asymptomatic, few patients may present with hematuria, lumbar pain, urinary tract infection, progressive increase in the number and size of the cysts and bleeding in the cyst [8]. The most life-threatening complication reported in ACKD is development of renal cell carcinoma (RCC). Screening is pivotal for the early detection of RCC and timely treatment leads to better patient survival [9]. The best screening tool for detecting cystic changes in native kidneys is ultrasonography. It is usually enough for the diagnosis but in certain conditions contrast tomography (CT) or magnetic resonance imaging (MRI) is required [10].

As internationally reported literature of ACKD in children is scarce and as per our knowledge, no regional pediatric study has been conducted on this subject. The purpose of this research is to describe the frequency of ACKD in children on chronic hemodialysis. This may help in surveillance and early identification of this entity and in turn reduce associated major and minor complications including life-threatening RCC.

## Materials and methods

This is a single-center cross-sectional study conducted at the hemodialysis unit and radiology department of Sindh Institute of Urology and Transplantation (SIUT) Karachi, Pakistan, from September 2020 to August 2021. The institutional ethical review committee approved (SIUT-ERC-2020/PA-231) the project and written informed consent was obtained from the parents/guardian.

Inclusion and exclusion criteria

Children with end-stage kidney disease (ESKD) diagnosed prior to 18 years of age and on maintenance hemodialysis twice a week for more than six months were enrolled in the study. Children with ESKD secondary to inherited or congenital cystic kidney disease were excluded.

Sample size calculation

The total population of ESKD children on hemodialysis for more than six months is 188 and based on the previous proportion of ACKD in children of ESKD is 21.6% with a margin of error of 3% and 95% confidence level. A total of 150 children were enrolled through the nonprobability consecutive sampling method [6].

Data collection

A detailed proforma was filled to include demographic, clinical, and radiological data. High-resolution ultrasound of the kidneys was done with a Toshiba Aplio i800 machine by the same consultant sonologist to reduce the observer bias and findings were recorded as per Bosniak Classification of cystic renal masses. None of our children fulfilled the class II or beyond therefore CT/MRI was not performed [11].

Operational definitions

Acquired Cystic Kidney Disease

ACKD was defined as the presence of three or more cysts in each kidney without a history of congenital or hereditary cystic kidney disease [12].

Solitary Cysts (SCs)

Less than three cysts in unilateral or bilateral kidneys are defined as SCs in our cohort without a prior history of congenital or hereditary cystic kidney disease.

Statistical analysis

The quantitative variables were expressed as mean/median with standard deviation (SD)/interquartile range (IQR) and qualitative variables as percentages. Means were compared using Student’s t-test. Categorical variables were assessed using the Chi-square test. The p-value equal to or less than 0.05 was considered significant.

## Results

A total of 150 children with ESKD were enrolled in the study with 63 (42%) males and 87 (58%) females. The mean age of the study participants was 14.5 ± 3.5 years (6-24) and the median duration of hemodialysis was 25 months (IQR= 7-216).

ACKD was found in 53 (35%) children, SCs in 18 (12%), and no cysts in 79 (53%) on ultrasonography. A higher frequency of ACKD was found in children more than 16 years of age 24 (45%), followed by 12 to 16 years 18 (34%) and less than 12 years 11 (21%), though results were insignificant (p-value 0.89). The rest of the characteristics of children with ACKD, SCs, and no cysts are shown in Table 1.

**Table 1:** Characteristics of patients ACKD; Acquired cystic kidney disease, SCs; Solitary cysts, CAKUT; Congenital anomalies of the kidney and urinary tract, GN; glomerulonephritis, CKD; Chronic kidney disease

	Total	ACKD	SCs	No cysts	P-value
Sample size	150	53	18	79	
Sex					
Male	63(42%)	22(41.5%)	10(55.5%)	31(39%)	0.44
Female	87(58%)	31(58.5%)	8(44.5%)	48(61%)	
Age (years)					
< 12	34(23%)	11(21%)	4(22.3%)	19(24%)	0.89
12-16	54(36%)	18(34%)	8(44.4%)	28(35%)	
>.16	62(41%)	24(45%)	6(33.3%)	32(41%)	
Etiology of ESKD					
CAKUT	23(15%)	8(15%)	4(22%)	11(14%)	0.45
Stones	31(21%)	9(17%)	2(11%)	20(25%)	
GN	23(15%)	6(11%)	5(28%)	12(15%)	
Unknown	64(43%)	27(51%)	7(39%)	30(38%)	
Others	9(6%)	3(6%)	0	6(8%)	
Solitary kidney	17(11%)	6 (35%)	2(12%)	9(53%)	0.99
CKD Duration (months)					
< 24 months	21(14%)	8(15%)	0	13(16%)	0.41
24-48 months	19(13%)	6(11%)	3(17%)	10(13%)	
> 48 months	41(27%)	12(23%)	8(44%)	21(27%)	
Unknown	69(46%)	27(51%)	7(39%)	35(44%)	

**Figure 1:**
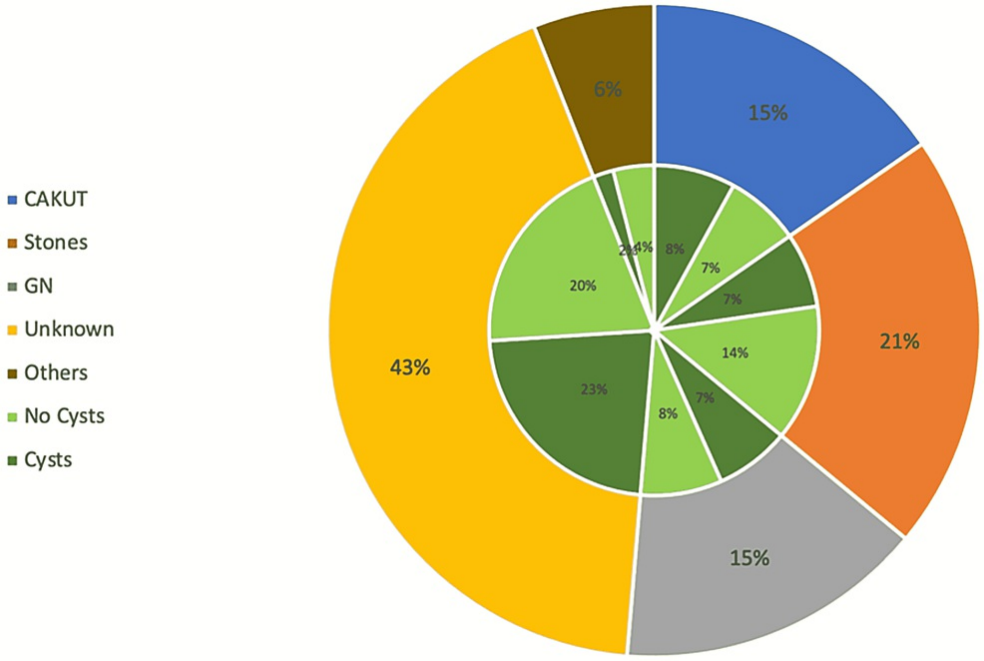
Frequencies of cystic kidney disease in different etiologies of end-stage kidney disease CAKUT; Congenital anomalies of kidney and urinary tract, GN; Glomerulonephritis

We did not find any correlation between the prevalence of cystic kidney disease and underlying etiology. However, a large number of ACKD 27 (51%) and SCs seven (39%) was found in patients with unknown etiology, which was not statistically significant (p-value 0.45). The causes of ESKD and the occurrence of cystic changes in our children are shown in Figure 1.

**Figure 2:**
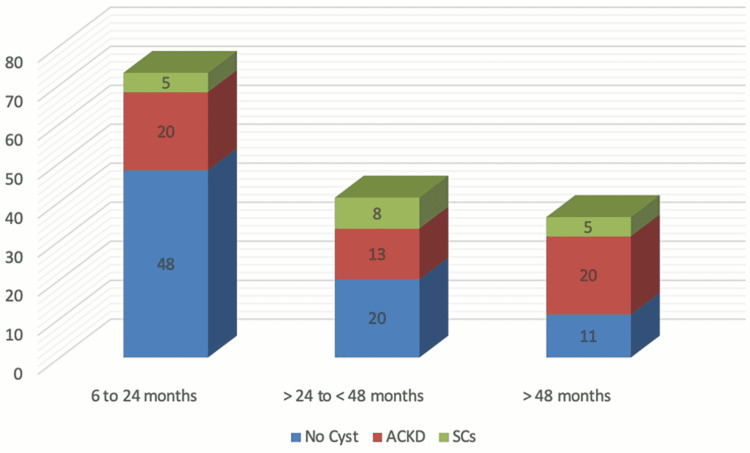
Association of cystic kidney diseases with duration of hemodialysis ACKD; Acquired cystic kidney disease, SCs; Solitary cysts

The solitary kidney was found in 17 (11%) of our patients and approximately eight (47%) children fulfilled the criteria for ACKD and SCs. A comparison of duration of dialysis and development of cysts revealed 20 (55%) ACKD in children who were on maintenance hemodialysis for more than 48 months. On the other hand, a lower frequency was observed at 20 (27%) in those with a duration of hemodialysis of fewer than 24 months, which is statistically significant (p-value 0.004) and is shown in Figure 2.

Cysts varied in size from median 0.7 cm (IQR=0.5-1.05) to 1 cm (IQR= 0.6-1.5). None of our patients experienced typical symptoms of flank pain, hematuria, and lower urinary tract. Similarly, we did not find complex cysts with septations or calcifications on sonography.

## Discussion

This is the largest single-center study on ACKD in children on chronic hemodialysis. Since nearly all renal transplantation in Pakistan is dependent on a live-related donor, a significantly large population of the patients remains on long-term maintenance dialysis.

Our study revealed 35% ACKD and 12% SCs in our hemodialysis patients. Our results are significantly lower than those reported by Mattoo et al., which were 46% and 25% in ACKD and SCs, respectively. The possible reason for the higher percentage could be the slightly older age group and longer duration of hemodialysis. However, Kyushu et al. reported 30% and 16% in ACKD and SCs which is similar to our results. No correlation was found between the age and development of ACKD in our study population (p-value 0.89) and similar findings were seen in studies published in the late 1990s [13,14].

Our study has shown a male to female ratio of 0.7:1. The ratio of males with ESKD on hemodialysis is 1.3, which is higher than females and that could be the reason to see the increased prevalence of ACKD in males [15]. Since our cohort had more females than males, therefore it is possible that we did not find a correlation between gender and the prevalence of ACKD (p-value 0.44). We do not believe that gender is a significant risk factor for this disease as reported by Choyke et al.; however further studies with a bigger cohort are needed to establish this finding [16].

The etiology of ESKD has not changed in more than a decade in developing countries like Pakistan and India. In the current study, the unknown etiology (42%) was the leading cause of ESKD and it could be due to a lack of routine health checkups. The underlying etiology has no correlation (p-value 0.45) with the development of ACKD neither in our study nor in reported literature [17,18].

The development of cysts and the duration of dialysis are directly proportional. Precise timing for the radiological appearance of cyst needs serial monitoring which cannot be concluded from a cross-sectional study. Our study has demonstrated that 33 (63%) children on hemodialysis for more than two years had ACKD in comparison to 20 (37%) less than two years which is statistically significant (p-value 0.004). As the duration of hemodialysis increases from 15 to 49 months, a progressive rise in the number of ACKD cases was noted by Narasimhan et al. [19]. Another study has reported a 40% prevalence of ACKD at three years of RRT and 90% by 5-10 years [16].

There are multiple published reports of patients with a variable number of cysts unilateral or bilateral not fulfilling the criteria for ACKD on renal replacement therapy. In our study, SCs were found in 18 (12%) children and a higher frequency was reported by Querfeld et al. at 33% [20]. There is no evidence-based definition of solitary cysts in the literature and therefore we have taken an arbitrary cut off of less than three cysts in our cohort. The solitary kidney was found in 17 (11%) children and among them, seven (35%) had ACKD, and two (12%) as having SCs. Cysts in solitary kidneys have been described previously in a study from Michigan, USA, in which out of four patients two were classified as no cysts, and one each had SCs and ACKD [13].

Regardless of variable frequency of complications described in the literature fortunately we did not find any of them in our ACKD cohort. The frequency of renal tumors in ACKD is 3%-7%, which is three to six times higher than the general population. It is usually bilateral and common in males [21]. ACKD-associated RCC has been recently defined as a separate entity by International Society of Urological Pathology [22]. Hence, an international working group for imaging of cystic kidney disease has recommended yearly follow-up of children on dialysis with ultrasound for ACKD [23]. In 2020, KDIGO nephron-oncology controversy conference has highlighted to prioritize the research areas like cost and tools of screening for malignancy in CKD and establish the causal effect of ACKD and RCC [24].

Limitations and strength

This study has the largest cohort of children on single mode of renal replacement therapy. Renal ultrasonography is used as a screening tool for the detection of ACKD but this modality has relatively low sensitivity for the detection of tiny cysts than CT scan. The causal effect of development of cysts and duration of disease cannot be ascertained through cross sectional study design and it needs prospective cohort studies.

## Conclusions

We have found a significant percentage of ACKD in prevalent hemodialysis children, which progressively increases with the duration of hemodialysis. There is utmost need for a consensus statement regarding the definition of ACKD in children from pediatric nephrology societies and leading registries. A strategy for surveillance and monitoring is required for the early detection and timely management to prevent devastating complications.
